# Regorafenib plus FOLFIRI with irinotecan dose escalated according to uridine diphosphate glucuronosyltransferase 1A1genotyping in previous treated metastatic colorectal cancer patients:study protocol for a randomized controlled trial

**DOI:** 10.1186/s13063-019-3917-z

**Published:** 2019-12-19

**Authors:** Cheng-Jen Ma, Tsung-Kun Chang, Hsiang-Lin Tsai, Wei-Chih Su, Ching-Wen Huang, Yung-Sung Yeh, Yu-Tang Chang, Jaw-Yuan Wang

**Affiliations:** 1 Division of Digestive and General Surgery, Department of Surgery, Kaohsiung Medical University Hospital, Kaohsiung Medical University, Kaohsiung, Taiwan; 2 Division of Colorectal Surgery, Department of Surgery, Kaohsiung Medical University Hospital, Kaohsiung Medical University, No. 100, Tzyou 1st Road, Kaohsiung, 807 Taiwan; 3 Graduate Institute of Clinical Medicine, College of Medicine, Kaohsiung Medical University, Kaohsiung, Taiwan; 4 Department of Surgery, Faculty of Medicine, College of Medicine, Kaohsiung Medical University, Kaohsiung, Taiwan; 5 Division of Trauma and Surgical Critical Care, Department of Surgery, Kaohsiung Medical University Hospital, Kaohsiung Medical University, Kaohsiung, Taiwan; 6 Division of Pediatric Surgery, Department of Surgery, Kaohsiung Medical University Hospital, Kaohsiung Medical University, Kaohsiung, Taiwan; 7 Graduate Institute of Medicine, College of Medicine, Kaohsiung Medical University, Kaohsiung, Taiwan; 8 Center for Cancer Research, Kaohsiung Medical University, Kaohsiung, Taiwan

**Keywords:** Regorafenib, *UGT1A1*, FOLFIRI, Dose escalation, Metastatic colorectal cancer

## Abstract

**Background:**

Regorafenib is an oral multikinase inhibitor for metastatic colorectal cancer (mCRC) previously treated with fluoropyrimidines, irinotecan, oxaliplatin, monoclonal antibodies targeting vascular endothelial growth factor, and monoclonal antibodies targeting epidermal growth factor receptor. A dose reduction from 160 mg to 120 mg regorafenib reduces regorafenib-associated adverse events (AEs). Dose adjustment of irinotecan in a 5-fluorouracil/leucovorin/irinotecan (FOLFIRI) regimen on the basis of an individual uridine diphosphate glucuronosyl transferase 1A1 (*UGT1A1)* genotype provides optimal oncological outcomes with acceptable AEs. The aim of this study is to address the efficacy and safety of a dose-adjusted combination of regorafenib and FOLFIRI for patients with mCRC.

**Methods:**

A prospective, multicenter, randomized in a 2:1 ratio, controlled, clinical trial with two parallel arms will be conducted to compare irinotecan dose-escalated FOLFIRI according to *UGT1A1* genotyping plus 120 mg regorafenib with 120 mg regorafenib alone in previously treated patients with mCRC. The primary endpoint is progression-free survival, and the secondary endpoints are overall survival, disease control rate, time to progression, and duration of treatment. Safety assessments will also be recorded.

**Discussion:**

Dose adjustment for regorafenib and irinotecan makes treatment-related AEs tolerable and makes the concomitant treatment practicable. This study will provide initial evidence regarding the efficacy and safety of a new combination of chemotherapy and a targeted agent for mCRC.

**Trial registration:**

ClinicalTrials.gov, NCT03880877. Prospectively registered on 19 March 2019.

## Background

Regorafenib monotherapy is currently the third- or fourth-line standard of care for patients with metastatic colorectal cancer (mCRC) who have been previously treated with fluoropyrimidines, irinotecan, oxaliplatin, and monoclonal antibodies targeting vascular endothelial growth factor (VEGF), and monoclonal antibodies targeting epidermal growth factor receptor (EGFR) for those with *KRAS* wild-type cancers. Regorafenib targets and inhibits membrane-bound and intracellular receptor tyrosine kinases (RTKs) that are involved in signaling for oncogenesis, angiogenesis and proliferation of cancer [1]. The global CORRECT trial demonstrated that regorafenib monotherapy had significantly better progression-free survival (PFS; 1.9 versus 1.7 months, *P* < 0.001), overall survival (OS; 6.4 versus 5.0 months; *P* = 0.0052), and disease control rate (DCR; 41% versus 15%, *P* < 0.0001) in previously treated mCRC [2]. Similarly, the CONCUR study, which was conducted in Asians, also yielded significant differences in PFS (3.2 versus 1.7 months, *P* < 0.0001), OS (8.8 versus 6.3 months; *P* = 0.00016), and DRC (51.5% versus 7.4%, *P* < 0.0001) [3]. The most frequently encountered adverse event (AE) of regorafenib is hand–foot skin reaction (HFSR), which leads to dose reduction or interruption of treatment in response to the AE. Dose reduction of regorafenib to 120 mg reduces the severity of AEs and allows better patient tolerance and compliance with comparable oncological results [4].

Irinotecan is a prodrug that is converted to its active metabolite, 7-ethyl-10-hydroxycamptothecin (SN-38), which acts on topoisomerase I in vivo to interrupt DNA replication in cancer cells and induce cell death. SN-38 is metabolized and detoxified by glucuronidation of uridine diphosphate glucuronosyl transferase (UGT), mainly the *UGT1A1* isoenzyme in the liver, to SN-38G [5]. The differences in capacity of glucuronidation of *UGT1A1* is determined by the number of repeats in the TATA box on the *UGT1A1* promoter, with the most common *UGT1A1* allele with 6 TA repeats (UGT1A1*1, wild type) and a variant allele with 7 TA repeats (UGT1A1*28, mutant type). Individuals with UGT1A1*28 exhibit reduced *UGT1A1* transcription and expression and consequently reduced SN-38 glucuronidation and increased toxicities of irinotecan [6]. Polymorphisms of *UGT1A1* therefore decide the rate of SN-38 glucuronidation and the following effects on the pharmacokinetics and toxicities of irinotecan [7]. Accordingly, dose adjustment of irinotecan based on individual *UGT1A1* genotyping along with 5-fluorouracil (5-FU) and leucovorin (LV) (FOLFIRI regimen) obtains acceptable AEs along with optimal oncological outcomes [8].

Based on the current literature, concomitant use of chemotherapy and targeted agents in treating mCRC shows potential add-on benefits for tumor control in the front-line setting. In our previous preliminary observational study, in which regorafenib plus FOLFIRI with dose escalation according to *UGT1A1* genotype were administered to previously treated patients with mCRC, we revealed superior oncological effects over regorafenib monotherapy [9]. As the result, the current study aims to explore the efficacy and safety of regorafenib and FOLFIRI concomitant treatment with dose-adjusted irinotecan based on *UGT1A1* genotype in previously treated mCRC patients compared to those who receive regorafenib alone in a prospective, randomized, two-arm, controlled setting.

## Methods/design

### Trial design

**Fig. 1:**
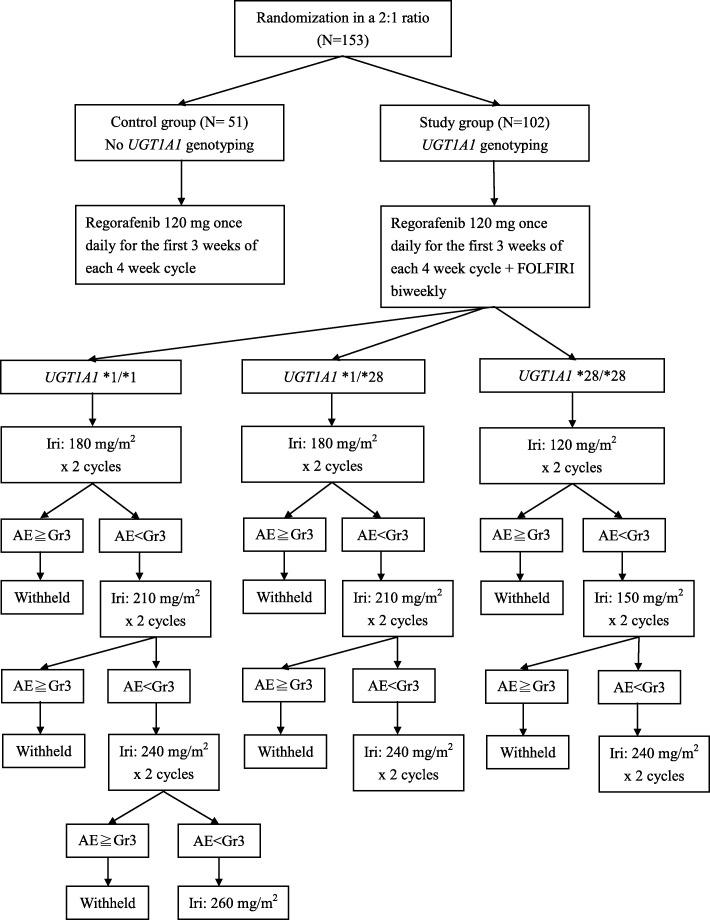
Flow diagram for this study. AE adverse event, FOLFIRI 5-fluorouracil/leucovorin/irinotecan, Gr grade, Iri irinotecan, UGT uridine diphosphate glucuronosyl transferase

This is a multicenter, randomized (in a 2:1 ratio) controlled clinical trial with two parallel arms, as summarized in Fig. 1. Neither the investigators nor the patients are masked to treatment allocation. In order to compare the efficacy and safety of regorafenib plus FOLFIRI with dose-adjusted irinotecan based on *UGT1A1* genotype (study group) and regorafenib monotherapy (control group), a total of 153 patients with mCRC that were previously treated with fluoropyrimidines, irinotecan, oxaliplatin, and monoclonal antibodies targeting VEGF, and monoclonal antibodies targeting EGFR for those with *KRAS* wild-type tumors, will be recruited, of which 102 participants will be assigned to the study group and 51 participants will be assigned to the control group in a 2:1 ratio.

### Study setting

The study will be conducted in four hospital centers in Taiwan.

### Study participants

Participants will be recruited from Kaohsiung Medical University Hospital, Cathay General Hospital, Taichung Veterans General Hospital and Taipei Veterans General Hospital by the colorectal surgeons. The planned recruitment period is 24 months and informed consent will be obtained from all participants before randomization.

### Eligibility criteria

#### Inclusion criteria

The following inclusion criteria will be used:

1. Cytologically/histologically confirmed mCRC
2. Patients who have been previously treated with, or are not considered candidates for, other locally approved standard treatment(s) and for whom the decision has been made per investigator’s routine treatment practice to prescribe regorafenib as third-line (*RAS* mutant) or fourth-line (*RAS* wild-type) therapy
3. Aged no less than 20 years at the time of acquisition of informed consent
4. Eastern Cooperative Oncology Group performance score 0–1
5. Patients with measurable or nonmeasurable disease in the colon or rectum, according to Response Evaluation Criteria in Solid Tumors (RECIST) version 1.1
6. Life expectancy of more than 12 weeks
7. Women with childbearing potential must agree to perform adequate contraception measures from informed consent until a least 12 weeks after the last study drug administration; the investigators or designee are requested to advise the patient to achieve adequate birth control
8. Adequate organ and bone marrow function as defined below:
  - Total bilirubin ≤1.5 × upper limit of normal (ULN)
  - Alanine aminotransferase and aspartate aminotransferase ≤2.5 × ULN (≤5 × ULN for patients with liver metastases)
  - Alkaline phosphatase ≤2.5 × ULN (≤ 5 × ULN for patients with liver metastases)
  - Amylase and lipase ≤1.5 × ULN
  - Serum creatinine ≤1.5 × ULN
  - Glomerular filtration rate ≥30 ml/min/1.73 m^2^, according to the modified diet in renal disease abbreviated formula
  - International normalized ratio/partial thromboplastin time ≤1.5 × ULN
  - Platelet count ≥100,000/mm^3^
  - Hemoglobin level ≥9 g/dL
  - Absolute neutrophil count ≥1500/mm^3^
9. Ability to understand and willingness to sign the written informed consent form

#### Exclusion criteria

The following exclusion criteria will be used:

1. Treatment with any other investigational agents within 28 days prior to enrollment in this study
2. Patients who have previously received FOLFIRI with irinotecan dose escalation
3. Other concurrent cancer or prior treatment for other carcinomas within the last 5 years, except curatively treated nonmelanoma skin cancer, superficial bladder tumors, and cervical cancer in situ
4. Extended field radiotherapy within 28 days or limited radiotherapy within 14 days prior to randomization
5. Major surgery within 28 days prior to the start of study treatment (diagnostic biopsy, laparotomy and line placement is not considered as major surgery)
6. Uncontrolled intercurrent illness including, but not limited to, ongoing or active infection, symptomatic congestive heart failure, myocardial infarction in the past 12 months, active gastrointestinal bleeding, central nervous system disorders or psychiatric illness/social situation that would limit compliance with study requirements or judged to be ineligible for the study by the investigator
7. History of myocardial infarction, deep venous or arterial thrombosis, or cerebrovascular accident during the last 6 months
8. Uncontrolled cardiac arrhythmias, unstable angina, or new-onset angina within 3 months prior to study entry
9. Uncontrolled hypertension despite optimal management (systolic blood pressure >150 mmHg or diastolic pressure >90 mmHg)
10. Patients with known central nervous system metastases
11. Having received any investigational agents or participated in any investigational drug study within 4 weeks prior to study enrollment
12. Pregnant or breast-feeding female (a pregnancy test must be performed on all female patients who are of child-bearing potential within 7 days of treatment initiation, and the result must be negative)
13. Inability to take oral medication
14. Poor compliance as judged by the investigator

### Genotyping

DNA is extracted from 4 mL of peripheral blood of patients with a PUREGENE® DNA isolation kit (Gentra Systems, Inc., Minneapolis, MN, USA). The genomic DNA extracted is then analyzed using direct sequencing to determine the *UGT1A1* promoter genotype. The primers are designed by primer 3 free software (http://primer3.wi.mit.edu), and the sequence of the forward primer is 5-AGT-CACGTGACACAGTCAAACA-3 and the reverse primer is 5-CTTTGCTCCTGCCAGAGGTT-3. The polymerase chain reaction (PCR) volume is 40 μL and the PCR conditions for the *UGT1A1* are as follows: 94.0 °C for 5 min; annealing for 20 s at 67.5 °C; primer extension for 20 s at 72.0 °C; and final extension for 10 min at 72.0 °C. A fragment analysis of the PCR products is conducted to verify the genotypes using the automated capillary electrophoresis on the ABI PRISM 310 Genetic Analyzer (Applied Biosystems, Foster City, CA, USA), and the genotypes are analyzed using GeneScan and Genotyper software (Applied Biosystems) [10].

### Interventions

#### Control group

The treatment for the control group will be oral regorafenib monotherapy at the dosing schedule of 120 mg once daily for the first 3 weeks of each 4-week cycle, without *UGT1A1* genotyping. If any regorafenib-related AEs greater than grade 3 develop, regorafenib will be stopped until the encountered AEs recover and the dose of regorafenib will be reduced by 40 mg daily.

#### Study group

The treatment for the study group will be oral regorafenib 120 mg once daily for the first 3 weeks of each 4-week cycle as described above and additional infusion of a FOLFIRI regimen. If any regorafenib-related AEs greater than grade 3 develop, regorafenib will be stopped until the encountered AEs recover and the dose of regorafenib will be reduced by 40 mg daily. According to the *UGT1A1* genotypes, the study group is further divided into the three following subgroups.

##### Subgroup 1: UGT1A1*1/*1 genotype

Oral regorafenib will be administered at a dose of 120 mg once daily for the first 3 weeks of each 4-week cycle. A FOLFIRI regimen will be initiated at a dose of 180 mg/m^2^ irinotecan and 200 mg/m^2^ LV intravenous (IV) infusion over 2 h followed by 5-FU (2800 mg/m^2^ as an IV infusion over a 46-h period). If no FOLFIRI-related AEs greater than grade 3 are observed after two cycles of each dose of irinotecan, the dose of irinotecan will be escalated in steps of 30 mg/m^2^. If any FOLFIRI-related AEs greater than grade 3 are encountered, the dose of irinotecan will be reduced by 30 mg/m^2^ or back to the previous dose and be held at that dose at the next cycle. The maximum dose of irinotecan for subgroup 1 is 260 mg/m^2^.

##### Subgroup 2: UGT1A1*1/*28 genotype

Oral regorafenib will be administered at a dose of 120 mg once daily for the first 3 weeks of each 4-week cycle. A FOLFIRI regimen will be initiated at a dose of 180 mg/m^2^ irinotecan and 200 mg/m^2^ LV IV infusion over 2 h followed by 5-FU (2800 mg/m^2^ as an IV infusion over a 46-h period). If no FOLFIRI-related AEs greater than grade 3 are observed after two cycles of each dose of irinotecan, the dose of irinotecan will be escalated in steps of 30 mg/m^2^. If any FOLFIRI-related AEs greater than grade 3 are encountered, the dose of irinotecan will be reduced by 30 mg/m^2^ or back to the previous dose and be held at that dose at the next cycle. The maximal dose of irinotecan for subgroup 2 is 240 mg/m^2^.

##### Subgroup 3: UGT1A1*28/*28 genotype

Oral regorafenib will be administered at a dose of 120 mg once daily for the first 3 weeks of each 4-week cycle. A FOLFIRI regimen will be initiated at a dose of 120 mg/m^2^ irinotecan and 200 mg/m^2^ LV IV infusion over 2 h followed by 5-FU (2800 mg/m^2^ as an IV infusion over a 46-h period). If no FOLFIRI-related AEs greater than grade 3 are observed after two cycles of each dose of irinotecan, the dose of irinotecan will be escalated in steps of 30 mg/m^2^. If any FOLFIRI-related AEs greater than grade 3 are encountered, the dose of irinotecan will be reduced by 30 mg/m^2^ or back to the previous dose and be held at that dose at the next cycle. The maximal dose of irinotecan for subgroup 3 is 180 mg/m^2^.

#### Stopping treatment/other medications

All treatment will be stopped in the event of patient withdrawal, disease progression, or unacceptable AEs, which are defined as nonhematological grade 4 AEs, no recovery from grade 3 AEs after two consecutive dose reductions, or no recovery after a 2-week treatment delay.

Throughout the study period, participants should not receive other cytotoxic or biological treatments for mCRC. If concomitant treatments are considered necessary based on investigator discretion, the product package insert should be referenced for contraindications and all concomitant treatments should be recorded on the relevant case report form (CRF) page.

### Outcome measurements

#### Primary outcome

The treatment response will be radiologically assessed every 2 months with computed tomography or magnetic resonance imaging. Objective responses will be classified according to RECIST and optimal treatment responses will be recorded. The primary endpoint is PFS, which is defined as the time from initiation of study treatment to first radiological progression or tumor-related death, whichever comes first.

#### Secondary outcome

Secondary outcomes will be as follows:

1. OS is defined as the time from initiation of study treatment to death due to any cause
2. DCR is defined as the percentage of patients whose best response was not progressive disease (i.e., complete response, partial response or stable disease)
3. Time to progression is defined as the time (in days) from the start of study treatment to the first documented disease progression
4. Duration of treatment is defined as the time interval from the start of study treatment to the day of permanent discontinuation (including death); mean dose and reasons for treatment discontinuation or dose modification will be recorded

### Safety assessment

The treatment-associated AEs will be assessed and recorded in each cycle using Common Terminology Criteria for Adverse Events version 5.0.

### Safety stopping rules

The study will be stopped according to the will or condition of the patients as judged by the investigators, or if any grade 4 AE develops.

### Participant timeline

The study is planned to start in May 2018 and is expected to end in May 2020, and will last for 2 years including recruitment, follow-up, data collection and data analysis. The schedule of study visits and assessments is summarized in Table 1.

**Table 1:** Schedule of visits and assessments

Assessment/procedure*	Enrollment	Allocation	Clinical regimen		Clinical tumor assessment	Completion/early termination visit	Survival follow-up
Time point	–2 week	0	Every 2 weeks	Every 4 weeks	Every 8 weeks		Every 8 weeks
Eligibility screen	X						
Informed Consent		X					
Randomization		X					
Demographic and medical history	X						
Cancer treatment history	X						
Tumor assessment: carcinoembryonic antigen, and computed tomography or magnetic resonance imaging	X				X	X	X
Eastern Cooperative Oncology Group performance status	X		X			X	
Urinalysis	X		X			X	
Hematology	X		X			X	
Clinical chemistry	X		X			X	
Creatinine clearance (calculated)	X		X				
Physical examination and vital signs	X		X			X	
Weight and height	X		X				
Concomitant medications	X		X			X	
Adverse events	X		X	X	X	X	X
Study drug administration			X	X			
Survival and tumor status/other anticancer treatment							X

*Available data will be collected; no additional diagnostic or monitoring procedures shall be applied to the patients other than routine clinical practice

### Sample size estimation

Using a per-protocol two-sided α of 0.025, a 2:1 randomization between regorafenib plus FOLFIRI dose escalation and regorafenib, and a median PFS of 3.2 months in the regorafenib group [9], the study would have 90% power to detect assumed median PFS of 7.0 months with regorafenib plus FOLFIRI [3]. The assumption of overall probability of events is 0.77 (10 progressions at the end of the study out of 13 in total) [9]. The calculated sample size is 121 patients. Considering a 20% drop-rate, a total of 153 patients should be enrolled into the study, with 102 assigned to the study group (FOLFIRI plus regorafenib group) and 51 to the control group (regorafenib monotherapy group).

### Recruitment

Participants will be enrolled from patients with mCRC whose diseases are refractory to fluoropyrimidines, irinotecan, oxaliplatin, and monoclonal antibodies targeting VEGF, and monoclonal antibodies targeting EGFR for those with *KRAS* wild-type cancers.

### Assignment of interventions

Before study initiation, a randomization sequence is computer generated (GraphPad statistical software, GraphPad, USA) and participants are allocated to either the study group or the control group in a 2:1 ratio in blocks of size 6 by an independent investigator.

### Data collection, management, and analysis

#### Data collection methods

Table 1 shows the details of data collection throughout the study period.

#### Data management

An electronic CRF will be made available to ensure data accuracy, completeness, and reliability. The research staff will preserve documented data from all sources on the CRF.

#### Statistical methods

Results will be compared using the Mann–Whitney *U* test or the Kruskal–Wallis test for categorical unpaired data. The Wilcoxon rank-sum test or the Friedman test will be used to analyze paired data. The Fisher’s exact test will be used to compare dichotomous variables. The Pearson chi-square test will be used to analyze nominal variables. The McNemar test will be used to analyze paired categorical data. The means will be compared using the two-sample test and analysis of variance or linear regression, as appropriate. However, for all aforementioned inferential analysis methods, the center effect will not be considered when comparing one treatment with another. Therefore, analysis of variance incorporating a center effect and the Cochran–Mantel–Haenszel test stratified by the center effect will be applied to replace the two-sample *t* test and the Fisher’s exact test. For efficacy analyses and part of the safety analyses (including laboratory data and vital signs data), and considering the effect of baseline data on the end points, analysis of covariance will be applied when comparing one treatment mean with another, with their respective baseline as covariates. Baseline data are defined as the data obtained before the first administration of treatment before surgery. End points are defined as the net change of post-treatment data from baseline data. Statistical analyses will be conducted using SPSS 20.0 (SPSS, Chicago, IL, USA). *P* values less than 0.05 will be considered statistically significant.

#### Data monitoring

All aspect of the study will be conducted under International Conference on Harmonization guidelines, Good Clinical Practice (GCP) guidelines, and government regulations. Monitoring (by telephone, fax, or on site) will be done by a representative of the study monitor designated by the investigators. The monitor will check CRFs for completeness, and clarify and crosscheck them with source documents. A Data Safety Monitoring Committee (DSMC) organized by the Institutional Review Board of Kaohsiung Medical University Hospital, Kaohsiung Medical University, Kaohsiung, Taiwan, will also monitor patient safety and treatment efficacy data while the study is ongoing. The DSMC is composed of two oncologists, a physician, and a statistician, none of whom is involved in the study.

The DSMC receives reports every 6 months to ensure that the investigation is conducted according to protocol design and regulatory requirements. The DSMC also monitors AEs/SAEs, and is involved in the issue of the risk–benefit to consider termination of the study.

#### Interim analysis

Early safety analysis will be performed 6 months after study is initiated.

#### Adverse events

Details of all AEs, including local and systemic reactions, will be collected including the event description, time of onset, clinicians’ assessment of severity, relation to study drugs and time of resolution or stabilization of the event. All AEs will be followed up until adequate resolution is seen. Pre-existing conditions will be recorded as medical histories. If the pre-existing condition does not change, it will not be recorded as an AE. However, if it deteriorates at any time during the study it will be recorded as an AE.

#### Auditing

Domestic authorities and the Institutional Review Board may request access to all source documents, CRFs, and other study documentation for on-site audit or inspection. The purpose of the audits is to evaluate study conduct and compliance with the protocol, standard operating procedures, GCP, and the applicable regulatory requirements. The observations and findings will be documented.

#### Consent

The free, informed and written consent of all participants will be collected by the investigator or a doctor representing them prior to final inclusion in the study. A copy of the information and consent form signed by both parties will be given to the patient; the investigator will keep the original.

#### Confidentiality

Only the patient number will be recorded in the CRF. The investigators maintain a personal patient identification list (patient numbers with corresponding patient names) to enable records to be identified.

#### Publication plan

The results of the trial will be published in international oncological, medical and scientific journals and presented at national and international congresses. The investigators will follow the rules and guidelines of the International Committee for Medical Journal Editors for authorship.

## Discussion

RTKs, such as VEGF receptor, EGFR, fibroblast growth factor receptor, platelet-derived growth factor receptor, RET and KIT, are responsible for several biological signaling pathways involved in the regulation of angiogenesis, oncogenesis and the tumor microenvironment [1]. Regorafenib is a small molecule that binds to and inhibits RTKs and therefore has antineoplastic activities. It is for this reason that regorafenib also shows various AEs, including HFSR, hypertension, thrombocytopenia, proteinuria, diarrhea, mucositis, hepatoxicity and so on. The most frequent grade 3 or higher AE related to regorafenib is HFSR, which results in dose reduction or interruption of treatment [2] and may limit the oncological efficacy of regorafenib. The occurrence of HFSR is even higher in the Asian population [11]. Modification of the dosing schedule (a change from a 160-mg dose given once daily, 21 days on, 7 days off, to 120 mg daily, 28 days on with the same accumulated dose of 3360 mg in a cycle) still has high rates of grade 3 HFSR although this recovers sooner than with the standard dosing schedule [9]. A Japanese study in which a dose reduction from 160 mg to 120 mg for 3 weeks in a 4-week cycle largely decreased the occurrence of grade 3 HFSR (75.0% in the 160-mg group and 16.7% in the 120-mg group) with comparable DCR (60.0% in the 160-mg group and 58.3% in the 120-mg group) [4]. As a result, the dose of regorafenib administered in the present study will be 120 mg once daily for the first 3 weeks in each 4-week cycle.

*UGT1A1* activity is the main factor determining cytotoxicity and AEs of irinotecan, and the US Food and Drug Administration has approved *UGT1A1* genotyping to predict irinotecan-induced severe diarrhea and neutropenia [12]. According to the *UGT1A1* genotype, an optimal dose of irinotecan can be achieved with balanced efficacy and toxicity for individuals. Lu et al. demonstrated a prognostic advantage of irinotecan dose escalation in an observational study on the basis of *UGT1A1* genotyping for mCRC treated with FOLFIRI plus bevacizumab [8], and an ongoing prospective, randomized controlled clinical trial is being conducted to examine the efficacy and safety of the aforementioned treatment [10].

In our preliminary, retrospective and observational study, in which 41 patients with mCRC were treated with regorafenib plus irinotecan dose-escalated FOLFIRI as a third- or fourth-line setting, median PFS was 6.0 months, median OS was 12.0 months and DCR was 58.5% [13]. Compared to the CORRECT and CONCUR studies, the oncological outcomes were satisfactory and treatment-related AEs were acceptable. To the best of our knowledge, there is no prospective study concerning the reintroduction of irinotecan for patients with mCRC; reintroduction of dose-escalated irinotecan for those previously treated with no irinotecan escalation may show similar results.

This is a multicenter, randomized clinical trial. There are no randomized clinical trials concerning the combination of regorafenib and irinotecan dose-escalated FOLFIRI for mCRC launched so far. Although there are aforementioned strengths to this study, this study is based on the results of a previous small, retrospective analysis and the rates of AEs of such a combination lead to some weaknesses.

This trial combines regorafenib, a targeted agent, with irinotecan dose-escalated FOLFIRI that may provide an alternative salvage treatment for previously treated mCRC.

### Trial status

The trial is currently recruiting; the first patient was recruited on 1 April 2019. Recruitment will be approximately completed before 31 March 2021 and the trial is estimated to end in October 2021. The current protocol is version 1.0, created on 1 February 2019. Any noncompliance with the trial protocol, GCP or manual of procedures requirements will be addressed in study subject source documents and correction actions and/or protocol modifications will be developed and implemented promptly. All protocol modifications will be approved by the Institutional Review Board and communicated to investigators and trial registries.

## Supplementary information

**Additional file 1.** SPIRIT 2013 checklist: recommended items to address in a clinical trial protocol and related documents.

## Data Availability

The anonymized participant data will be available for other researchers to apply for use 1 year after publication on request. Written proposals will be assessed by members of the Trial Steering Committee and a decision will be made about the appropriateness of the use of data. A data sharing agreement will be put in place before any data will be shared. The institution where this trial is conducted must be cited in any publication.

## Abbreviations

5-FU: 5-Fluorouracil
AE: Adverse event
CRF: Case report form
DCR: Disease control rate
DSMC: Data Safety Monitoring Committee
EGFR: Epidermal growth factor receptor
FOLFIRI: 5-Fluorouracil/leucovorin/irinotecan
GCP: Good Clinical Practice
HFSR: Hand–foot skin reaction
IV: Intravenous
LV: Leucovorin
mCRC: Metastatic colorectal cancer
OS: Overall survival
PCR: Polymerase chain reaction
PFS: Progression-free survival
RECIST: Response Evaluation Criteria in Solid Tumors
RTK: Receptor tyrosine kinase
SN-38: 7-Ethyl-10-hydroxycamptothecin
UGT: Uridine diphosphate glucuronosyl transferase
ULN: Upper limit of normal
VEGF: Vascular endothelial growth factor
